# Mind the gap: examining policy and social media discourse on Long COVID in children and young people in the UK

**DOI:** 10.1186/s12889-025-22563-0

**Published:** 2025-04-12

**Authors:** Macarena Chepo, Sam Martin, Noémie Déom, Ahmad Firas Khalid, Cecilia Vindrola-Padros

**Affiliations:** 1 School of Nursing, Universidad Andrés Bello, Santiago, Chile; 2 Department of Targeted Intervention, University College London (UCL), Charles Bell House 43 - 45 Foley Street, London, W1W 7TY UK; 3 Oxford Vaccine Group, Churchill Hospital, University of Oxford, Oxford, UK; 4 Centre for Implementation Research, Canadian Institutes of Health Research Health System Impact Fellowship, Ottawa Hospital Research Institute, Ottawa, ON Canada

**Keywords:** Long COVID, Children and young people (CYP), COVID- 19, UK health policy, Social media discourse analysis

## Abstract

**Background:**

Long COVID in children and young people (CYP) has posed significant challenges for health systems worldwide. Despite its impact on well-being and development, policies addressing the needs of CYP remain underdeveloped. This study examines UK Long COVID policies using ethical frameworks, integrating policy and social media analyses to explore public and professional concerns.

**Methods:**

A mixed-methods approach was applied. Policy documents were reviewed using Thompson et al.'s pandemic preparedness framework and Campbell and Carnevale’s child-inclusive ethical model. Social media discourse (12,650 posts) was analysed using Brandwatch™ to identify key themes around CYP and Long COVID policies. Data was collected and triangulated through the LISTEN method, which integrates policy analysis with social media discourse to ensure a holistic understanding of systemic gaps and public perceptions.

**Results:**

Analysis highlighted gaps in accountability, inclusiveness, and transparency in policy development. Social media data reflected significant public dissatisfaction, primarily critiquing government accountability (90% of posts) and delayed policy responsiveness (29% of posts). Key ethical challenges included limited CYP representation and unequal access to services.

**Conclusions:**

Recommendations include improving transparency, incorporating CYP perspectives in policymaking, and ensuring equitable access to care. These findings provide a foundation for ethically sound and inclusive policies addressing Long COVID in CYP.

**Supplementary Information:**

The online version contains supplementary material available at 10.1186/s12889-025-22563-0.

## Background

The term ‘Long COVID’ – along with its variations, including ‘*long term COVID- 19*’, ‘*long-haul COVID*’ or ‘*Post COVID- 19 condition’* – first emerged in April 2020. It appeared in online patient narratives shared on social media, grassroots initiatives led by patient activist and early publications by a small group of researchers. In the latter half of 2020, key international bodies, including the World Health Organization (WHO) and the Centre for Disease Control and Prevention (CDC), officially recognised Long COVID as a significant health concern [1]. In February 2021, Anthony Fauci further recognised Long COVID by defining it as ‘post-acute sequelae of COVID’ (PASC) [2].

Despite growing research, scientists and healthcare professionals still lack a full understanding of the long-term consequences of COVID- 19 [3]. While studies of potential PASC symptoms are increasingly being published, researchers and healthcare professionals continue to stress the importance of redefining Long COVID as new symptoms are identified [4, 5]. Consequently, policymakers are continuously working to define and frame the issue of Long COVID to ensure it can be effectively addressed [6, 7]. 

Understanding Long COVID in children and young people (CYP) is critical, as their developmental stages, immune responses, and social environments differ significantly from adults. This creates unique symptom patterns and long-term health impacts, emphasising the need for tailored medical and policy interventions that address the developmental and social needs of CYP. By July 2022, the WHO had aligned its definition of PASC in CYP with that of adults but acknowledged that “the frequency and characteristics of [PASC] are still under investigation” [3, 8, 9].

In February 2023, a clinical case definition detailed the specific symptoms for Post COVID- 19 condition in CYP, including fatigue, altered smell, and anxiety [10]. These findings aimed to unify understanding and guide governments and health organisations toward informed policy decisions [10].

At the time of writing, policies addressing PASC in CYP primarily focus on adapting healthcare services to meet paediatric needs [11]. Although recent findings from the UK-based children & young people with Long COVID (CLoCK) study reveal that CYP with PASC experience diminished quality of life and well-being [12], policies to address these challenges have not yet been implemented.

This policy review addresses the following research question: “How have UK healthcare policies addressed Long COVID in CYP between 2020–2022, and to what extent do these policies align with Thompson et al.'s and Campbell and Carnevale's frameworks for ethical pandemic response?”.

The study has four key objectives:

1. To analyse UK healthcare policies related to Long COVID in CYP from 2020–2022
2. To evaluate how these policies incorporate CYP perspectives
3. To examine public discourse around these policies through social media analysis
4. To identify gaps and make recommendations for future policy development

The review uses an adapted version of Thompson et al.’s framework, as developed by Campbell and Canaverale, to identify policies aimed at implementing and facilitating Long COVID healthcare delivery for Children and Young People (CYP) in the UK. Thompson et al.'s framework is based on the idea that pandemic preparedness requires a multi-faceted and ethical approach that spans all stages of pandemic planning: prevention, preparedness, response, and recovery [13]. While the framework highlights the importance of stakeholder collaboration, evidence-based decision-making, and continuous learning and adaptation, Campbell and Carnevale and others have criticised its limited inclusion of CYP, particularly in applying policy to CYP in the context of the COVID- 19 pandemic and PASC [14–16]. The advocacy for the inclusion of CYP also aligns with the Convention for the Rights of the Child (CRC) [17]. The CRC, an international treaty adopted by the United Nations in 1989, establishes the rights of individuals under 18, including the right be heard, protection from discrimination, and the right to survival and development. However, these rights are often overlooked in policymaking for Long COVID, as highlighted in this study.

To address this gap, we have used the adapted model of Thompson’s et al.’s framework by Campbell and Carnevale [15] (Appendix 1: Table 1). This adaptation integrates five key processes – accountability, inclusiveness, openness and transparency, reasonableness, and responsiveness – to ensure policies are ethically sound and equitable. Accountability ensures that policymakers are held responsible for outcomes, while inclusiveness emphasises engaging CYP as stakeholders in decision-making. Openness and transparency build public trust by clearly communicating policy decisions, reasonableness ensures policies are evidence-based and proportional, and responsiveness allows for timely adjustments based on new data and feedback.

Additionally, this study applies the LISTEN method [18] to operationalise these frameworks. Developed by the Rapid Research Evaluation and Appraisal Lab (RREAL) [18], the LISTEN method is designed for collaborative and digital analysis of large qualitative datasets in time-sensitive contexts. By combining traditional qualitative techniques with advanced digital tools (e.g., text network analysis), the method ensures efficiency, inclusivity, and rigour. It is particularly suited for mixed-methods research like this study, as it enables triangulation between formal policy analysis and real-world social media discourse. This approach ensures that insights reflect both systemic perspectives and stakeholder voices.

The utilisation of the frameworks of Thompson et al. and Campbell & Carnevale, alongside the LISTEN method, provide a robust foundation for understanding the ethical dimensions of policymaking during pandemics. Thompson’s emphasis on prevention, preparedness, response, and recovery ensures a systematic approach, while Campbell & Carnevale’s adaptation incorporates a child-inclusive lens, addressing the specific needs of CYP. By integrating these frameworks, this study seeks to evaluate not only the content of policies but also their ethical alignment, highlighting gaps in accountability, inclusiveness, and transparency. The LISTEN method further operationalises this ethical lens, allowing the triangulation of policy analysis with real-world social media discourse to validate findings and inform recommendations.

## Methods

### Study design and conceptual framework

Using the LISTEN method [18], we employed a mixed-methods design, combining a rapid policy review with a quantitative–qualitative social media analysis. We used Thompson et al.’s [13] ethical pandemic preparedness model as a starting point, which emphasises prevention, preparedness, response, and recovery in an evidence-based, iterative manner. Building on critiques [15], we incorporated an adapted child-inclusive framework, focusing on accountability, inclusiveness, openness and transparency, reasonableness, and responsiveness in policy decisions (see Appendix 1).

In this study, the LISTEN method enabled the integration of data from two distinct sources: UK policy documents and social media discourse. The method supported the triangulation of findings, ensuring that both sources informed and validated each other.

### Policy review

Policies were identified using systematic searches of UK governmental and health agency databases.

Inclusion criteria focused on Long COVID guidelines for CYP published between 2020 and 2022 (see Appendix 2: Table 3).

#### Data sources

We searched for policy documents, guidelines, and relevant governmental announcements in publicly available databases (UK Government, NHS, Public Health England, NIHR). We included documents published from March 2020 to December 2022 that addressed “Long COVID,” “post-acute coronavirus syndrome,” or “PASC,” specifically referencing CYP.

#### Selection and extraction

Two researchers independently screened documents according to our inclusion criteria. Discrepancies were resolved via discussion. For each included policy, we extracted the date of publication, publishing institution, target population, and recommendations.

#### Analysis

We coded the content in relation to Thompson et al.’s pandemic preparedness elements and the additional child-focused processes [15]. Framework analysis [19] was then used to categorise each policy guideline by how it addressed CYP within an ethical and child-rights perspective.

### Social media analysis

Twitter (now X) was selected as the primary social media platform for its widespread use in public health discourse and its ability to capture real-time, dynamic conversations. Brandwatch™, a social listening tool, was used to capture relevant data due to its advanced features, including geographical and timeframe restrictions, as well as Boolean search terms. This approach facilitated the identification of tweets discussing policies related to Long COVID and the lived experiences of CYP.

#### Data sources

Tweets were collected from users in the UK between January 2021 and February 2023 using advanced Boolean search terms referencing Long COVID in CYP (Appendix 3). These terms were designed to ensure comprehensive data collection, capturing discussions on policy and political discourse specific to CYP and Long COVID.

#### Sampling and data cleaning

Two researchers independently screened the dataset for relevance, removing irrelevant or duplicate posts. This process yielded 12,650 tweets mentioning policy or political discourse. A random 15% sample (*n* = 1,898) was extracted for qualitative review to balance analytical depth and manageability while ensuring thematic representativeness. Following additional screening to ensure alignment with the research question, a final dataset of 1,629 tweets was retained for analysis. Discrepancies during selection were resolved through discussion.

#### Analysis

Tweets were analysed using an iterative coding process, categorising them into the five child-inclusive ethical processes outlined in the adapted framework. Descriptive analytics, such as word clouds, were used to identify key themes, hashtags, and user concerns. Quantitative measures were applied to assess the frequency of each category, and overlaps were noted to highlight interconnections between themes.

### Validation and triangulation

To ensure rigour and consistency in our mixed-methods approach, we employed an iterative triangulation process, synthesising findings from the policy review and social media analysis. The LISTEN method facilitated this integration by providing a structured framework for combining diverse data sources, ensuring that insights from one dataset informed and validated those from the other. Additionally, the LISTEN method was instrumental in mapping ethical principles such as inclusiveness and responsiveness onto the triangulation process, ensuring that both policy analysis and social media discourse were evaluated against these principles. For instance, inclusiveness was assessed by coding both policy content and public discourse for evidence of CYP engagement, while responsiveness was analysed through the timeliness of interventions and feedback loops evident in the data.

The policy review process involved three stages of intercoder reliability checks. For initial coding, one researcher (ND) conducted a first round of coding, identifying key themes related to the ethical processes (e.g., accountability, inclusiveness). A second researcher (MC) reviewed and expanded the coding, ensuring that overlooked or ambiguous themes were captured. This iterative process included discussions to resolve discrepancies. For the final validation, the research team (SM, ND and MC) conducted an intercoder reliability meeting where the team reviewed all codes and queries and achieved a final agreement threshold of 80%. This collaborative approach ensured alignment across all interpretations.

For the social media analysis validation, a similar intercoder reliability process was applied. For the initial categorisation of social media posts, two researchers (SM and MC) independently coded a subset of tweets using the child-inclusive ethical framework. Key themes were identified, such as accountability, responsiveness, and inclusiveness. Both researchers then met to reconcile any discrepancies in their coding. Any unresolved themes were discussed with ND to ensure consistency and clarity. The final validation of the coding framework was carried out by a third researcher (FK) to ensure robustness and minimise bias. Agreement across the team again reached 80%, confirming reliability.

The triangulation process then synthesised findings from the policy review and social media analysis, ensuring that both datasets complemented and validated one another. This iterative approach, facilitated by the LISTEN method, allowed us to integrate top-down (policy-driven) and bottom-up (community-driven) insights, providing a comprehensive understanding of the ethical challenges and gaps in addressing Long COVID in CYP.

Themes identified in the policy review were systematically cross-referenced with those emerging from social media discourse. For example, the absence of child-friendly guidelines highlighted in the policy review was reflected in social media critiques about the inaccessibility of information for CYP and their caregivers. Similarly, concerns about the uneven distribution of paediatric clinics, identified as a policy gap, were echoed in tweets expressing frustration over regional inequities in access to care.

Through this integration, the triangulation process ensured that the analysis captured both systemic policy-level shortcomings and the lived experiences and perceptions of stakeholders. This comprehensive synthesis allowed for actionable recommendations grounded in both evidence-based policy analysis and real-world social insights.

### Ethical considerations

In line with University College London Research Ethics Committee guidelines [20], this study adhered to strict ethical standards for internet research. Only publicly available tweets were included in the analysis, and all data were anonymised to prevent identification of individuals. Personal identifiers, including usernames, were excluded, and no direct quotes that could be traced back to users were used in this paper. These measures ensured compliance with ethical guidelines for the use of social media data, focusing on privacy and the secure handling of publicly accessible information.

## Results

To ensure a structured presentation of the study's findings, the results are organised as follows. First, the context of Long COVID in CYP is introduced, accompanied by an overview of the key policies and measures identified through a review of government and health agency databases in the UK. This is followed by an analysis of these findings using the ethical pandemic preparedness framework proposed by Thompson et al. Subsequently, relevant tweets are examined to capture public perceptions of Long COVID policies. Finally, the insights from the policy review and social media analysis are integrated, offering a comprehensive synthesis of the issue. This structured approach aims to provide a holistic and empirically grounded analysis that informs more inclusive and ethically robust policy strategies.

### Policy landscape: evolving recognition of CYP needs

Policy analysis revealed an incremental acknowledgment of the unique challenges faced by CYP with Long COVID. Early policies, such as the NHS post-COVID assessment clinics established in December 2020, focused on adult pathways without addressing paediatric needs [21]. It was not until June 2021 that the UK announced 15 dedicated paediatric post-COVID clinics, following increasing pressure from patient advocacy groups and emerging data highlighting the disproportionate impact of Long COVID on CYP. This timeline highlights a significant delay in addressing CYP-specific requirements, leading to prolonged unmet healthcare needs and delayed policy adjustments of emerging evidence.

Despite increasing recognition of “children’s rights” in later policies, the CRC was not explicitly referenced. This omission underscores Campbell and Carnevale’s [15] critique that healthcare poles often fail to prioritise the rights of CYP. Although the National Institute for Health and Care Research (NIHR)’s 2022 guidelines acknowledge the importance of “listening to children’s voices” in mental health research [22], they stopped short of incorporating the CRC as a guiding framework (see Appendix 4: Table 4). Such gaps in policy alignment may further delay progress in addressing the developmental, psychological, and social impacts of Long COVID on CYP.

Analysis of the social media data further shows that there was a push for the inclusion of CYP’s lived experiences in policymaking (44 tweets, 2.7% of the data), particularly emphasising the psychological and social impacts of long COVID that policies often neglect. This discourse highlights growing awareness to create equitable and effective healthcare strategies. However, the implementation of such principles remains inconsistent. This inconsistency is exemplified by the delayed roll-out of paediatric clinics and the ongoing lack of comprehensive data collection on CYP with Long COVID. These gaps underscore the need for stronger policy frameworks that integrate children’s rights and voices.

The LISTEN method’s integration of social media analysis revealed significant gaps in the practical application of the ethical frameworks. For example, while policies emphasised accountability mechanisms for adult services, they failed to implement similar measures for CYP-specific interventions, as evidenced by public critiques on social media. Similarly, the absence of CYP voices in policymaking, reflected in both the policy review and social discourse, underscores the need for stronger inclusiveness and transparency mechanisms. This alignment between systemic shortcomings and public sentiment reinforces the validity of the findings.

### Application of ethical processes to CYP in Long COVID policies

The adapted ethical framework revealed notable gaps across the five processes, reflecting systemic shortcomings in addressing the needs of CYP with long COVID.

- 1. Accountability
- Policies outlined accountability mechanisms for adult services, such as audits for post-COVID clinics, but lacked clear evaluation strategies for paediatric services. Additionally, stakeholder oversight for CYP services was limited, and no specific metrics were provided to assess the effectiveness of educational adaptations or mental health interventions for Long COVID affected CYP.
- 2. Inclusiveness
- Decision-making processes often relied on indirect input from parents and carers, rather than consulting CYP directly. Social analysis also revealed advocacy for incorporating CYP’s experiences into policymaking. This omission contrasts with international best practices that prioritise youth participation in health policymaking [15]. The exclusion of CYP voices risks neglecting their unique perspectives on the physical, emotional, and social impacts of Long COVID.
- 3. Openness and transparency
- Government documents related to Long COVID policies were publicly available, but they were not designed to be accessible to CYP. No youth-friendly materials were created to ensure understanding, nor were any efforts made to translate complex medical and policy jargon into formats appropriate for younger audiences. This lack of accessibility diminishes CYP understanding and engagement with Long COVID policies, leaving them unaware of available resources or services.
- 4. Reasonableness
- Policies acknowledged the wide-ranging symptoms of Long COVID, they failed to account for its long-term developmental, educational, and psychological impacts on CYP. For instance, there was no consideration of how Long COVID might affect academic performance, social development, or transitions to adulthood for affected youth. This oversight is particularly concerning given the critical developmental stages CYP experience, during which prolonged illness can have far-reaching consequences.
- 5. Responsiveness
- The establishment of tailored paediatric clinics was an important step toward addressing the needs of CYP with Long COVID. however, the rollout of these clinics lagged behind adult services Additionally, these clinics were geographically concentrated in the south of England, exacerbating regional health inequities and limiting access for CYP in underserved areas.

### Social media discourse: strong calls for accountability and responsiveness

Social media analysis of 1,629 relevant tweets provided insight into public perceptions of Long COVID policies. The findings show a growing public concern around the ethical aspects of policymaking, particularly focusing on accountability and responsiveness.

#### Accountability (90% of tweets (n = 1,151))

Posts frequently criticised the government for inadequate investment in paediatric Long COVID research and inconsistent school safety measures. For instance, one user tweeted: “*How can they ignore the data? Children with Long COVID deserve better. Where’s the funding for proper research?*” (summarised for ethical reasons). Such posts underscore the perceived disconnect between public demand and governmental priorities, reflecting frustration with a lack of transparency and perceived inequities in policy focus.

#### Responsiveness (29% of tweets (n = 361))

Discussions highlighted delays in establishing paediatric clinics and frustration over limited government response to CYP-specific concerns. Parents often expressed feeling neglected, emphasising at government action only came after intense advocacy efforts gained media attention. This theme illustrates broader concerns about the timeliness of healthcare service adjustments meet CYP needs, as policies often lagged behind emerging evidence.

#### Inclusiveness, openness and reasonableness (9% of tweets (n = 117))

Although less frequently mentioned, these themes overlapped with calls for accountability. Several tweets noted the lack of CYP representation in policymaking processes, while other tweets criticised the absence of clear, child-friendly communication in policy implementation This lack of inclusiveness further exacerbated public mistrust in governmental handling of Long COVID in children.

Analysis of the tweets reveals mistrust in the government’s handling of Long COVID policies for CYP. As shown in Appendix 1: Table 2, many users highlighted the failure to incorporate CYP’s voices in policymaking, with a small but notable proportion of parents explicitly referencing the CRC or child rights to argue that children should not simply treat CYP as “mini-adults.” These tweets underline the pressing need for consistent stakeholder engagement, clear communication, and the ethical inclusion of CYP perspectives in policy development and implementation.

### Integration of policy and social media findings

The triangulation of policy analysis and social media discourse revealed critical overlaps, gaps, and contradictions in the UK’s response to Long COVID in CYP.

#### Delayed paediatric interventions

Policy timelines demonstrated slow responsiveness in addressing CYP-specific needs, with significant delays in establishing paediatric-specific clinics. Social media critiques mirrored these findings, with advocacy groups and affected families expressing frustration at the reactive nature of policies. Furthermore, recent findings from the CLoCK study, the largest matched cohort study of Long COVID in CYP, underscore the significant prevalence of persisting symptoms such as tiredness and shortness of breath among CYP who tested positive for SARS-CoV- 2. Specifically, 39% of test-positive participants reported tiredness three months post-infection, with symptoms persisting or increasing up to 12 months [23].This alignment highlights the importance of integrating CYP-specific requirements into pandemic preparedness plans rather than relying on retrospective adjustments.

#### Regional inequities

Both policy analysis and social media posts revealed significant disparities in the availability of paediatric Long COVID clinics across different regions. Families in underserved areas frequently posted about the challenges of accessing care, often emphasising longer wait times and financial burdens related to travel. These regional inequities reflect systemic issues in resource allocation and the need for targeted interventions to ensure equitable access to healthcare services.

#### CRC and child-friendly communication

While policies made rhetorical references to “children’s voices”, they fell short of embedding meaningful structural inclusion of CYP perspectives in policy communications. Social media users emphasised the need for structural inclusion of CYP perspectives, such as child-friendly materials and clearer communication strategies to engage CYP directly. The closure of the “Your COVID Recovery” website further exacerbated this gap, as the platform had provided accessible information for adolescents. This disconnect between policy intent and execution illustrates the need to align initiatives with the United Nations CRC [17], ensuring that CYP are not only heard but actively involved in shaping policies affecting their lives.

**Fig. 1: Timeline of key UK policy developments for Long COVID in children and young people (CYP):**
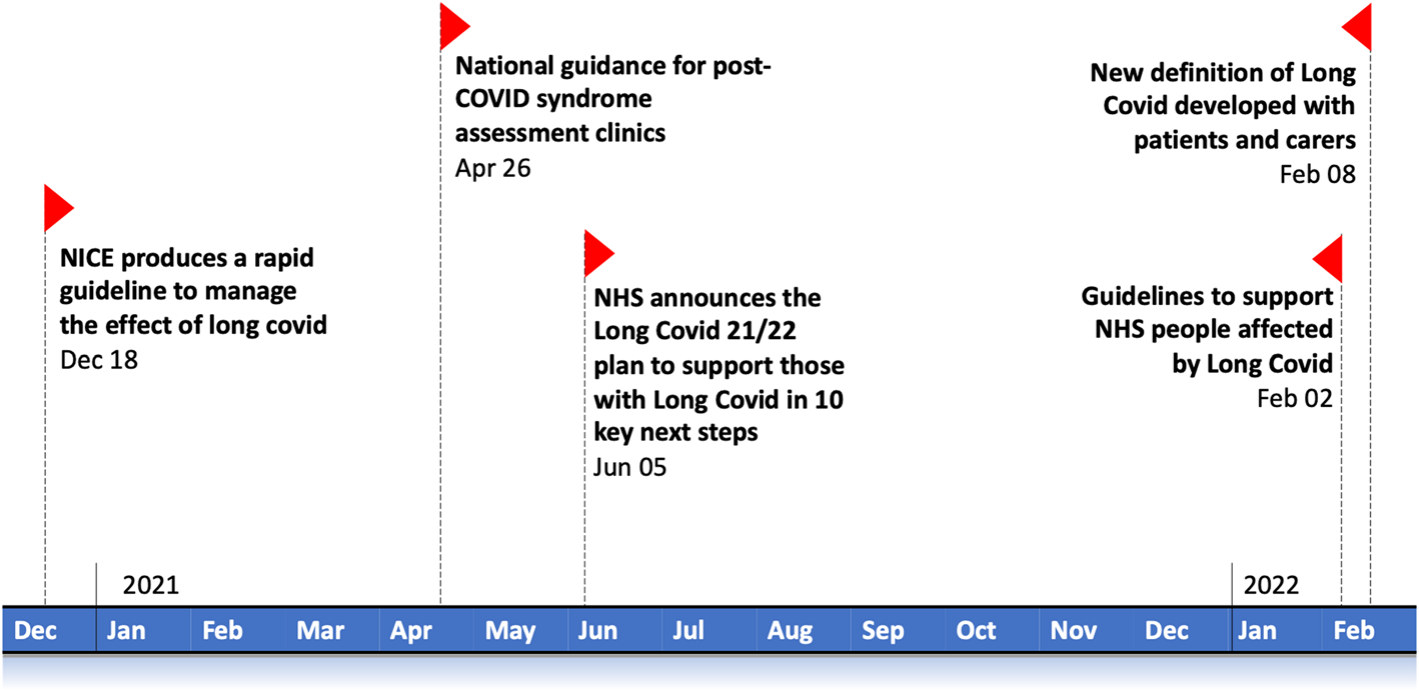
.

Figure 1 shows an Infographic summarising the UK policy timeline during the COVID- 19 pandemic. It highlights significant milestone for CYP, beginning with the initial establishment of adult-oriented post-COVID clinics in December 2020, the delayed announcement of paediatric clinics in June 2021, and later updates tailored to children’s healthcare needs. The now closed “Your COVID Recovery” website is also marked as a resource that demonstrated the potential of youth-centred communication but was discontinued, leaving an unaddressed gap in support for CYP.

## Discussion

This study offers critical insights into the evolution of UK Long COVID policies for CYP and their alignment with ethical frameworks for pandemic response. By applying the LISTEN method to triangulate policy analysis and social media discourse, we identified significant ethical and practical challenges in addressing CYP needs during the COVID- 19 pandemic. This study sought to answer three key research questions:

1. What ethical principles underpin UK Long COVID policies for CYP?
2. How do public and professional discourses reflect these principles and identify gaps in policy responsiveness?
3. How can policy frameworks better incorporate CYP perspectives to promote equity and accountability?

Our findings provide critical insights into how UK Long COVID policies for CYP align with ethical frameworks for pandemic response, reflecting both progress and persistent challenges.

### Policy evolution and ethical challenges

Our analysis of UK Long COVID policies reveals incremental recognition of CYP-specific needs, yet persistent ethical shortcoming–particularly in accountability, inclusiveness, and transparency. Initially, policies adapted adult-focused frameworks, delaying the development of tailored intervention for CYP. While the rollout of paediatric-specific clinics in 2021 marked progress, disparities in regional access and the absence of formal consultation mechanisms with CYP limited the inclusiveness and equity of these policies.

These findings align with Baz et al., who highlight how barriers to Long COVID care disproportionately impact vulnerable groups, including CYP [24]. Similarly, a comparative analysis of European policies reveals inconsistencies in addressing children's needs, with delayed policy responses and limited consultation mechanisms across multiple countries [25].

Using Thompson et al.’s framework, complemented by Campbell and Carnevale’s child-inclusive model, we identified key gaps:

- **Accountability**: Mechanisms to monitor and evaluate the impact of paediatric interventions remain limited. This aligns with Campbell and Carnevale’s critique that pandemic responses often marginalise CYP.
- **Inclusiveness**: Minimal direct engagement with CYP in policymaking, relying instead on indirect inputs from caregivers, has hindered the representation of their lived-experiences.
- **Transparency**: The absence of youth-friendly communication materials have undermined CYP’s ability to engage and understand policies affecting their health.
- **Responsiveness and Reasonableness**: Evidence-based interventions tailored to CYP were slower to materialise compared to adult-focused policies, reflecting a systemic delay in recognising paediatric needs.

These findings underscore the need for ethically grounded approaches that prioritise equity and accountability in policymaking for CYP during public health crises. For example, incorporating mandatory audits of paediatric interventions could enhance accountability.

### Integration of public and professional discourses

Social media analysis offered a valuable lens to understand public perceptions of Long COVID policies. Accountability emerged as the most frequently discussed theme (90% of tweets), with significant concerns about the government’s failure to prioritise CYP needs. The theme of responsiveness (29%) highlighted widespread dissatisfaction with the government’s prioritisation of CYP needs. Additionally, themes of inclusiveness and transparency emphasised the exclusion of CYP voices from decisionmaking processes and the lack of accessible information tailored to young audiences.

By triangulating these insights with policy findings, we uncovered overlaps and contradictions between policy intentions and public perceptions. Public discourse critiqued the government’s stated commitment to inclusiveness, pointing out the exclusion of CYP perspectives. While policies prioritised paediatric clinic rollouts, public concerns about regional inequities and delays in implementation remained unaddressed.

These results highlight the importance of integrating stakeholder feedback into policy development. As Thompson et al. [17] emphasise, transparency and stakeholder engagement are critical to building trust and ensuring policies address the needs of marginalised groups. The study’s findings reinforce the critical need to embed ethical principles, such as inclusiveness and accountability, into pandemic policymaking. For example, the LISTEN method revealed that delays in the rollout of paediatric clinics were not only logistical but also reflected a lack of responsiveness to emerging evidence. By aligning recommendations with Campbell & Carnevale’s emphasis on child-inclusive processes, this study demonstrates that meaningful CYP engagement is essential for ensuring policies are equitable and effective. For instance, structured youth advisory boards could provide direct input into policymaking processes. Similarly, the triangulation approach provided by the LISTEN method highlights the importance of integrating public and professional discourses to address policy shortcomings.

### Practical barriers and recommendations for inclusion

In addition to ethical challenges, this study identified practical barriers that hindered the implementation of effective Long COVID policies. Regional disparities in clinic availability, workforce shortages, and limited funding emerged as critical obstacles. Addressing these barriers. requires actionable strategies to enhance both ethical and practical dimensions of policymaking.

To improve accountability, policymakers should establish robust monitoring mechanisms, such as regular audits, third-party reviews, and feedback loops for CYP and their caregivers. For example, regular reporting on paediatric clinic outcomes could ensure transparency and build public trust.

Inclusiveness can be enhanced through deliberative dialogues that convene all stakeholders, including CYP, caregivers, and healthcare providers, to co-develop policies. Transparency should be prioritised by providing clear, accessible updates on policy decisions and outcomes, ensuring CYP and their families are well-informed.

Finally, addressing practical barriers necessitates equitable resource allocation to reduce regional disparities, investment in workforce development, and integration of mental health support into Long COVID care pathways. Policymakers must also develop clear school mitigation plans informed by stakeholder input to ensure policies support the holistic needs of CYP with Long COVID.

### Policy implications

This study leveraged the LISTEN method in order to generate evidence-based policy recommendations. The integration of policy analysis and social media discourse provides a a comprehensive understanding of the systemic and stakeholder-driven gaps in current Long COVID policies for CYP. Each recommendation presented here aligns with the ethical principles of equity, inclusiveness, accountability, and responsiveness, offering actionable pathways to improve clinical practice, operational policy, and strategic planning.

#### Enhancing clinical practice

The results highlight the critical need for CYP-specific diagnostic and treatment protocols. Our analysis revealed that current guidelines, which often adapt adult-centric models, may not adequately consider the developmental variability and unique symptom profiles of CYP, particularly in mental health support pathways. Standardisation of clinical assessments tailored to CYP, incorporating both physical and psychological assessments, and updating these protocols based on emerging evidence are urgent priorities. This is further reinforced by stakeholder frustrations expressed in social media data, which emphasised gaps in mental health services for CYP.

Mental health support pathways emerged as a particularly fragmented area of care, risking exacerbation of Long COVID’s psychological impact on CYP. Embedding mental health services within Long COVID care frameworks–including routine screenings and clear referral mechanisms to specialist support– would significantly strengthen the quality of care. These measures align with broader calls for family-centred approaches, recognising the interconnected needs of affected children and their families.

#### Addressing regional inequities in service access

One of the most pronounced themes in both policy review and social media discourse was the inequitable distribution of paediatric Long COVID clinics, with services disproportionately concentrated in southern England. This geographical disparity highlights a systemic failure to meet the principles of equity and inclusiveness, as identified in ethical frameworks and our triangulated findings. While a strategic expansion of paediatric services is essential, this could take the form of a phased approach prioritising regions with the most care deficits.

Variations in the availability of paediatric Long COVID services reflect broader trends observed in European policies, where mitigation strategies and healthcare access differed significantly between countries and regions [25]. This geographical disparity exacerbates health inequalities, with children from lower-income backgrounds facing additional barriers to accessing care [24].

Virtual care models, such as telemedicine, may complement physical clinics by improving access for families in remote areas. However, challenges such as digital connectivity and caregiving training must be addressed to ensure equitable implementation. Although direct evidence on the success of telemedicine was limited in this study, existing literature supports its role in mitigating access barriers for underserved populations. The re-opening of the (now closed) “YourCovidRecovery” website could be part of this strategy.

#### Embedding inclusiveness in policy design

Inclusiveness emerged as a significant ethical and practical gap in our analysis, with both policies and social media critiques highlighting the limited involvement of CYP in decision-making. For instance, our analysis revealed that social media posts called for the inclusion of CYP voices in policy development, underlining the absence of formal consultation mechanisms. While some policies referenced the importance of “children’s voices,” the lack of formal consultation mechanisms for CYP arguably diminishes the effectiveness of these efforts.

One recommendation to address this gap would be the establishment of structured youth advisory boards and feedback channels. These mechanisms could involve collaboration with schools, community organisations, or healthcare providers, ensuring diverse participation from CYP across different socioeconomic and geographical contexts. Digital tools could also play a role in broadening engagement, enabling CYP from underserved regions to contribute remotely. Such measures allow CYP to engage meaningfully, with age-appropriate, culturally sensitive tools that would ensure diverse perspectives are represented.

Moreover, addressing inclusiveness must also involve a review of the CRC to identify overlooked rights and disparities across groups of CYP. These disparities are often compounded by regional inequities in service access, which intersect with other forms of marginalisation, such as socioeconomic status, ethnicity, and rurality. For instance, children from lower-income households in remote areas may face additional challenges, such as transportation costs or limited digital connectivity, further hindering access to care. Addressing these inequities demands a commitment to a health equity framework, where resource allocation is tailored to the needs of diverse and underserved populations.

Inclusiveness is also closely tied to other ethical principles, such as transparency and accountability. For example, incorporating CYP perspectives into policymaking fosters public trust and ensures that policies reflect the lived realities of those they are designed to serve. By embedding inclusiveness into policy design, policies can align with the ethical principles that underpin equitable healthcare systems.

#### Strengthening transparency and accountability

Transparency and accountability were recurring themes in our analysis, reflecting a gap between policy intentions and stakeholder perceptions. Social media data highlighted frustrations with inconsistent government communication and the absence of clear timelines for policy implementation. For example, stakeholders expressed concerns about the delayed rollout of paediatric services, as highlighted in both policy review findings and social media critiques.

Regular evaluations of service outcomes, supported by robust audit frameworks, are critical to addressing these gaps. These evaluations could include monitoring clinic performance metrics, such as patient wait times, service utilisation rates, and patient satisfaction surveys, to ensure consistent care quality. Additionally, audits could assess the equity of service distribution by examining geographic, socioeconomic, and demographic disparities in access to care. Involving independent reviewers in these audits would enhance impartiality and public trust. Regular publication of these evaluations in accessible formats would further promote transparency, ensuring that stakeholders are informed about progress and challenges.

Engaging stakeholders—particularly CYP, their families, and healthcare providers—could further strengthen these efforts. Stakeholders can be involved in co-developing evaluation frameworks, participating in feedback sessions, and contributing to the interpretation of findings**.** This participatory approach aligns with principles of inclusiveness and accountability, helping to rebuild public trust in policymaking.

#### Adopting a health equity framework

The findings also call for a broader integration of health equity principles in policy development. Disparities in access to paediatric services, such as the concentration of clinics in Southern England, reflect systemic inequities that disproportionately affect marginalised CYP populations. These include low-income households, rural communities, and ethnic minorities, who may face compounded barriers such as transportation costs, digital connectivity limitations, and language barriers. The recent publication of the CLoCK study findings also highlighted potential disparities in symptom management and reporting between SARS-CoV- 2 positive and negative children, reflecting broader challenges in achieving equity and inclusiveness within the UK’s healthcare policy landscape for Long COVID [23].

Addressing these challenges requires the application of a robust health equity framework to ensure fairer resource allocation and the reduction of disparities.

Applying a health equity framework to service planning could help with fairer allocation of resources, prioritising underserved regions and communities. Community-based consultations could also be employed to gather insights directly from affected groups, ensuring that policy decisions reflect diverse lived experiences. These strategies would help to ensure that health policies are both equitable and responsive. Regular equity audits could also be conducted to evaluate access and outcomes, with adjustments made to address disparities. These audits can examine service accessibility, patient outcomes, and cultural and linguistic support, helping policymakers identify gaps and implement targeted solutions.

Such measures may prove critical for ensuring that CYP-specific policies uphold the ethical principle of reasonableness by addressing diverse needs comprehensively.

#### Investing in CYP-focused research

Finally, this study has shown the need for dedicated funding streams to support research on Long COVID in CYP. Potential avenues for funding include government initiatives, such as the NIHR in the UK, as well as collaborations with international organisations or public–private partnerships. Establishing sustained funding mechanisms is critical to building a robust evidence base for CYPspecific interventions, which remains a significant limitation in both policy development and clinical practice.

Additionally, involving CYP as co-researchers where appropriate would enhance the relevance and applicability of research findings, aligning with the ethical principles of inclusiveness and accountability. Participatory research methods, such as involving CYP in advisory roles or co-designing studies, would ensure their perspectives and lived experiences are directly incorporated into the research process. However, challenges such as ensuring ethical participation, providing adequate training, and avoiding tokenism must be addressed. Solutions may include creating age-appropriate research frameworks, offering capacity-building workshops, and facilitating ongoing engagement through accessible digital tools.

The limited evidence base for CYP-specific interventions arguably constrains both policy development and clinical practice. Prioritising research into the long-term impacts of Long COVID, including mental health, educational outcomes, and service evaluations, would provide the foundation for more effective and responsive policies.

### Limitations and future directions

This study provides valuable insights into the ethical and practical challenges of UK Long COVID policies for CYP. However, limitations should be acknowledged.

First, reliance on publicly available social media data limits the representativeness of stakeholder views, particularly excluding younger children and digitally marginalised populations or those less inclined to share their opinions. Future studies could address this by incorporating qualitative interviews or surveys to capture a broader range of perspectives.

Second, the LISTEN method’s reliance on comprehensive policy documents and metadata from social platforms makes it vulnerable to inconsistent policy transparency and algorithmic changes. Diversi Diversifying data sources, such as including parliamentary debates, healthcare provider insights, and CYP-focused workshops could strengthen the robustness of future analyses.

Third, this study’s UK-centric scope limits the generalisability of findings to other contexts. Comparative analyses of Long COVID policies across different countries could offer valuable insights into global best practices and innovative approaches.

Finally, the study highlights key evidence gaps that warrant further research, including regional disparities in access to CYP-specific care, the effectiveness of telemedicine for underserved populations, the intersection of Long COVID with socioeconomic and demographic factors, and the longitudinal impacts of Long COVID on CYP’s mental health, education, and development. Addressing these limitations through future research could significantly enhance the comprehensiveness and applicability of policy recommendations.

## Conclusion

This study provides a critical ethical assessment of UK Long COVID policies for children and young people (CYP), highlighting significant gaps in inclusiveness, equity, and transparency. By employing the LISTEN method, which integrates policy analysis and social media discourse, we were able to triangulate findings and generate evidence-based insights into public concerns and systemic shortcomings. These insights offer actionable pathways to refine policy and practice.

Our recommendations focus on enhancing clinical practice through CYP-specific diagnostic and treatment protocols, addressing regional inequities in service access, embedding inclusiveness in policy design, and strengthening transparency and accountability. Additionally, we emphasise the adoption of health equity frameworks and sustained investment in CYP-focused research to ensure the long-term success of policy interventions.

Limitations to this study must be acknowledged. Reliance on social media data may have excluded digitally marginalised populations, and the study’s dependence on publicly available policy documents and metadata could affect the reliability of analyses. Furthermore, the UK-centric scope limits the generalisability of findings to other contexts. To address these limitations, future research should incorporate diverse data sources, explore international comparative analyses, and investigate the longitudinal impacts of Long COVID policies on CYP's mental health, education, and development.

By addressing these gaps and prioritising the inclusion of CYP voices in policymaking, the adoption of robust equity frameworks, and the investment in targeted research, policymakers can lay the foundation for a more ethical, inclusive, and effective pandemic recovery. Immediate action is necessary to ensure these recommendations are implemented before the long-term impacts of inaction become more pronounced.

## Supplementary Information

Supplementary Material 1

## Data Availability

No datasets were generated or analysed during the current study.

## Abbreviations

CDC: Centre for Disease Control and Prevention
CLoCK: Children & Young People with Long COVID Study
COVID: Coronavirus Disease
CRC: Convention on the Rights of the Child
CYP: Children and Young People
GPs: General Practitioners
HCR: Health Care Readiness (as per WHO definitions)
IAPT: Improving Access to Psychological Therapies
LISTEN: Collaborative and Digital Analysis of Big Qualitative Data in Time Sensitive Contexts
MHPSS: Mental Health and Psychosocial Support
NHS: National Health Service
NICE: National Institute for Health and Care Excellence
NIHR: National Institute for Health and Care Research
ONS: Office for National Statistics
PASC: Post-Acute Sequelae of COVID
PHE: Public Health England
RCPCH: Royal College of Paediatrics and Child Health
UCL: University College London
UCLREC: University College London Research Ethics Committee
UK: United Kingdom
WHO: World Health Organisation

## References

[1] Miyake E, Martin S. Long COVID: Online patient narratives, public health communication and vaccine hesitancy. Digital Health. 2021;7:205520762110596. 10.1177/20552076211059649. 10.1177/20552076211059649 PMC8638072 34868622

[2] Taeschler P, Cervia C, Zurbuchen Y, Hasler S, Pou C, Tan Z, et al. Autoantibodies in COVID19 correlate with anti-viral humoral responses and distinct immune signatures. MedRxiv. 2022;2022.01.08.22268901. 10.1101/2022.01.08.22268901. 10.1111/all.15302 PMC9111424 35364615

[3] World Health Organization. Coronavirus update 54: Update on clinical long-term effects of COVID-19 [Internet]. 2021 Mar 26 [cited 2025 Jan 24]. Available from: https://www.who.int/docs/default-source/coronaviruse/risk-commsupdates/update54_clinical_long_term_effects.pdf.

[4] Gutiérrez-Canales LG, et al. Persistence of COVID-19 symptoms and quality of life at three and twelve months after hospital discharge. Medicina. 2024;60(6):944. 10.3390/medicina60060944. 38929561 10.3390/medicina60060944 PMC11205838

[5] Xuereb RA, et al. Long COVID syndrome: A case-control study. Am J Med. 2023. 10.1016/j.amjmed.2023.04.022. 37169323 10.1016/j.amjmed.2023.04.022 PMC10168190

[6] Fotheringham P, Harriott T, Healy G, Arenge G, Wilson E. Pressures and influences on school leaders navigating policy development during the COVID-19 pandemic. Br Educ Res J. 2022;48(2):201–27. 10.1002/berj.3760.

[7] World Health Organization. Health systems response to post-COVID-19 condition. World Health Organization; 2022 [cited 2025 Jan 24]. Available from: https://apps.who.int/iris/bitstream/handle/10665/366381/WHO-EURO-2023-664446410-67217-eng.pdf.

[8] Stephenson T. Expert testimony to inform NICE guideline development—Professor Sir Terence Stephenson [Internet]. National Institute for Health and Care Excellence (NICE); 2022 [cited 2025 Jan 24]. Available from: https://files.magicapp.org/guideline/58a5979d-20e94b8e-b6ae-10182bd6e9d2/files/FORM_Expert_testimony_Terence_Stephenson_v4_FINAL_r393383.pdf

[9] World Health Organization. Interim statement on COVID-19 vaccination for children [Internet]. 2021 Nov 24 [cited 2025 Jan 24]. Available from: https://www.who.int/news/item/11-08-2022interim-statement-on-covid-19-vaccination-for-children.

[10] Health Care Readiness team. A clinical case definition for post-COVID-19 condition in children and adolescents by expert consensus. World Health Organization; 2023 [cited 2025 Jan 24]. Available from: https://www.who.int/publications/i/item/WHO-2019-nCoV-PostCOVID-condition-CA-Clinical-case-definition-2023-1.

[11] Royal College of Paediatrics and Child Health. COVID-19 Recovery Committee (Scottish Parliament): Long COVID inquiry—consultation response [Internet]. 2023 Feb [cited 2025 Jan 24]. Available from: https://www.rcpch.ac.uk/resources/covid-19-recovery-committee-scottishparliament-long-covid-inquiry-consultation-response.

[12] Pinto Pereira SM, Shafran R, Nugawela MD, Panagi L, Hargreaves D, Ladhani SN, et al. Natural course of health and well-being in non-hospitalised children and young people after testing for SARS-CoV-2: A prospective follow-up study over 12 months. Lancet Reg Health Eur. 2023;25: 100554. 10.1016/j.lanepe.2022.100554. 36504922 10.1016/j.lanepe.2022.100554 PMC9719829

[13] Thompson AK, Faith K, Gibson JL, Upshur RE. Pandemic influenza preparedness: An ethical framework to guide decision-making. BMC Med Ethics. 2006;7(1):12. 10.1186/1472-69397-12. 10.1186/1472-6939-7-12 PMC1698926 17144926

[14] Campbell S, Cicero Oneto C, Saini MP, Attaran N, Makansi N, Passos Dos Santos R, et al. Impacts of the COVID-19 pandemic on children: An ethical analysis with a global-child lens. Glob Stud Child. 2021;11(1):105–14. 10.1177/2043610620976142.

[15] Campbell S, Carnevale FA. Children as an afterthought during COVID-19: Defining a childinclusive ethical framework for pandemic policymaking. BMC Med Ethics. 2022;23(1):126. 10.1186/s12910-022-00866-w. 36471326 10.1186/s12910-022-00866-w PMC9720957

[16] Sundaram N, Bonell C, Ladhani S, Langan SM, Baawuah F, Okike I, et al. Implementation of preventive measures to prevent COVID-19: A national study of English primary schools in summer 2020. Health Educ Res. 2021;36(3):272–85. 10.1093/her/cyab016. 33860299 10.1093/her/cyab016 PMC8083280

[17] UNICEF. Convention on the Rights of the Child [Internet]. [cited 2025 Jan 24]. Available from: https://www.unicef.org/child-rights-convention.

[18] Vera San Juan N, Martin S, Badley A, Maio L, Gronholm PC, Buck C, et al. Frontline healthcare workers' mental health and well-being during the first year of the COVID-19 pandemic: Analysis of interviews and social media data. J Med Internet Res. 2023;25:e43000. 10.2196/43000. 10.2196/43000 PMC10426381 37402283

[19] Gale NK, Heath G, Cameron E, Rashid S, Redwood S. Using the framework method for the analysis of qualitative data in multi-disciplinary health research. BMC Med Res Methodol. 2013;13:117. 10.1186/1471-2288-13-117. PMC3848812 24047204

[20] University College London. UCL Research Ethics (UCLREC): Research classed as exempt [Internet]. 2023 [cited 2025 Jan 24]. Available from: https://www.ucl.ac.uk/research-ethics/doi-need-ethical-approval#When_don’t_I_need_to_apply.

[21] NHS England. Children and young people with COVID. Your COVID Recovery [Internet]. 2022 Nov 9 [cited 2025 Jan 24]. Available from: https://www.yourcovidrecovery.nhs.uk/children-and-young-people-with-covid/

[22] National Institute for Health and Care Excellence (NICE), Scottish Intercollegiate Guidelines Network, Royal College of General Practitioners. COVID-19 rapid guideline: Managing the long-term effects of COVID-19 [Internet]. 2022 [cited 2025 Jan 24]. Available from: https://www.nice.org.uk/guidance/ng188/resources/covid19-rapid-guideline-managingthe-longterm-effects-of-covid19-pdf-51035515742.

[23] Rojas NK, De Stavola BL, Norris T, Cortina-Borja M, Nugawela MD, Hargreaves D, et al. Developing survey weights to ensure representativeness in a national, matched cohort study: Results from the children and young people with Long COVID (CLoCk) study. BMC Med Res Methodol. 2024;24(1):134. 38902672 10.1186/s12874-024-02219-0 PMC11188173

[24] Baz SA, Woodrow M, Clutterbuck D, Fang C, Mullard J, Banerjee A, et al. Long COVID and Health Inequalities: What’s Next for Research and Policy Advocacy? Health Expectations. 2024;27(5): e70047. 39358980 10.1111/hex.70047 PMC11447195

[25] Soriano-Arandes A, Brett A, Buonsenso D, Emilsson L, De La Fuente Garcia I, Gkentzi D, et al. Policies on children and schools during the SARS-CoV-2 pandemic in Western Europe. Front Public Health. 2023;25(11):1175444. 10.3389/fpubh.2023.1175444 PMC10411527 37564427

