# Supplementary material for: Mind the gap: examining policy and social media discourse on Long COVID in children and young people in the UK
Source: BMC Public Health. 2025 Apr 12;25:1373. doi: 10.1186/s12889-025-22563-0 (PMC11992780; doi:10.1186/s12889-025-22563-0)
Supplement: Supplementary file 1 — Supplementary Material 1 [file 12889_2025_22563_MOESM1_ESM.docx]

# Appendix 1

## Table 1. Theoretical Framework

| **Theoretical Concept (Campbell and Carnivale adaptations of the Thompson et. al model)** | **Definition** | **Application in Policy Review** | **Application in Social Media Analysis** |
| --- | --- | --- | --- |
| **Accountability** | Mechanisms to monitor pandemic-related decisions must prioritise and incorporate the rights of children into policy decisions and should assess the ways these rights are being overlooked. These assessments should be explicitly led by decision-makers, private organisations, legislative bodies, and other key stakeholders, ideally through a third-party review that ensures neutrality and fairness. | \| **Mentions of audits, oversight mechanisms, and policy evaluation measures were coded.** \| \| --- \|      \|  \| \| --- \| | Social media posts expressing concerns about the lack of government and healthcare system accountability were identified. |
| **Inclusiveness** | Young people must be considered stakeholders by decision-makers and society in order to be included. Their voices are often shared through the work of adults, but opportunities for direct engagement with young people (in ways that align with their interests) should be developed and implemented during pandemics. Fundamentally, these voices must be solicited and genuinely listened to in these settings as well, and intersectional elements of identity must be acknowledged and accounted for in engagement strategies.  The CRC should be reviewed and consulted to see where children’s rights are overlooked, how their lives are impacted by being excluded, and where disparities exist across groups of children. | References to public consultations, CYP advisory groups, and youth participation mechanisms in policy design were analysed. | Discussions about the exclusion of CYP from decision-making processes were examined, highlighting their lack of representation in Long COVID policies. |
| **Openness and Transparency** | Decisions must be clear and understandable for the entire population. Decisions that are publicly accessible—ones that promote ‘health policy literacy’—require the translation of decision trails into formats that allow understanding on the part of *all* young people too.  With transparency must come the possibility of criticism or suggestions from young people, as with any population, and a need for public bodies to respond to these perspectives. | Government documents and communication strategies aimed at CYP and their families were identified. | Posts highlighting the lack of clear or accessible information about service access and CYP rights were analysed. |
| **Reasonableness** | Qualitative research that engages populations of young people, and their families, should be considered reasonable evidence for policy decisions. Young people, themselves, should also be seen as reasonable and reliable.  Moreover, the scope of information being examined must also be broader. For example: many of the impacts affecting children are future-focused, though immediate impacts are prioritized in pandemic decision-making. Longer-term risks must be included in long-term pandemic plans.   While this focus on present concerns is understandable, one by-product is that it may lead policymakers to disregard the ways in which adapting solutions and redistributing resources now to mitigate predictable future harm (for the sake of children’s futures) can have both immediate and long-term benefits for all of society. | The scientific justifications for policy development, use of epidemiological data, and adaptation of protocols specifically for CYP were assessed. | Debates on social media regarding whether the measures taken were proportional to the severity of the issue in CYP were coded. |
| **Responsiveness** | We need to continue to ensure that we are responsive to the needs of young people too, especially as new information emerges about the ways they are being harmed by COVID-19 that may be outside the ‘transmission’ concerns.  A formal mechanism for parents, caregivers, and young people to provide feedback must be developed in a way that is attentive to the capacities of an organisation or state. | Adjustments and revisions to policies based on new research findings on Long COVID in CYP were analysed. | Criticism on social media regarding delays in policy implementation or failure to adapt to the evolving pandemic was identified. |

## Table 2. Application of Theoretical Framework in Data Coding

| **Theoretical Concept (Campbell and Carnivale adaptations of the Thompson et. al model)** | **Definition** | **Application in Policy Review** | **Application in Social Media Analysis** |
| --- | --- | --- | --- |
| **Accountability** | Mechanisms to monitor pandemic-related decisions must prioritise and incorporate the rights of children into policy decisions and should assess the ways these rights are being overlooked. These assessments should be explicitly led by decision-makers, private organisations, legislative bodies, and other key stakeholders, ideally through a third-party review that ensures neutrality and fairness. | \| Mentions of audits, oversight mechanisms, and policy evaluation measures were coded. \| \| --- \|      \|  \| \| --- \| | Social media posts expressing concerns about the lack of *government and healthcare system accountability were identified*.    Example:  *“The UK government has permanently failed people with #LongCovid. It has not helped in prevention or in the delivery of information on the impact of this disease, both in young and healthy people, as well as in thousands of patients with chronic diseases”* |
| **Inclusiveness** | Young people must be considered stakeholders by decision-makers and society in order to be included. Their voices are often shared through the work of adults, but opportunities for direct engagement with young people (in ways that align with their interests) should be developed and implemented during pandemics. Fundamentally, these voices must be solicited and genuinely listened to in these settings as well, and intersectional elements of identity must be acknowledged and accounted for in engagement strategies.  The CRC should be reviewed and consulted to see where children’s rights are overlooked, how their lives are impacted by being excluded, and where disparities exist across groups of children. | References to public consultations, CYP advisory groups, and youth participation mechanisms in policy design were analysed. | Discussions about the exclusion of CYP from decision-making processes were examined, highlighting their lack of representation in Long COVID policies.    Example:  *“Covid Modellers and MPs should actually be listening to parents and doctors. Seeing a child sick and suffering with Covid and then #LongCovid is very frightening for both the parent and child. Their voices need to be heard and reflected in policy.”* |
| **Openness and Transparency** | Decisions must be clear and understandable for the entire population. Decisions that are publicly accessible—ones that promote ‘health policy literacy’—require the translation of decision trails into formats that allow understanding on the part of *all* young people too.  With transparency must come the possibility of criticism or suggestions from young people, as with any population, and a need for public bodies to respond to these perspectives. | Government documents and communication strategies aimed at CYP and their families were identified. | Posts highlighting the lack of clear or accessible information about service access and CYP rights were analysed.    Example:  *"The unclear guidance from the UK Government is once again putting lives at risk, including children and adults who are susceptible to long-COVID. It's disappointing that the government has yet to update its guidance to allow remote learning options for families. Meanwhile, the UK's open borders policy continues to invite the worst variants from around the world to coexist with us”* |
| **Reasonableness** | Qualitative research that engages populations of young people, and their families, should be considered reasonable evidence for policy decisions. Young people, themselves, should also be seen as reasonable and reliable.  Moreover, the scope of information being examined must also be broader. For example: many of the impacts affecting children are future-focused, though immediate impacts are prioritized in pandemic decision-making. Longer-term risks must be included in long-term pandemic plans.   While this focus on present concerns is understandable, one by-product is that it may lead policymakers to disregard the ways in which adapting solutions and redistributing resources now to mitigate predictable future harm (for the sake of children’s futures) can have both immediate and long-term benefits for all of society. | The scientific justifications for policy development, use of epidemiological data, and adaptation of protocols specifically for CYP were assessed. | Debates on social media regarding whether the measures taken were proportional to the severity of the issue in CYP were coded.    Example:  *“It's important to understand that COVID-19 is not just another flu! It is a complex disease that affects multiple systems in the body, with potentially severe outcomes beyond hospitalisation or death. #LongCOVID, is a condition that can leave both adults and children struggling with debilitating symptoms, is one such outcome. Unfortunately, there is still much we don't know about the long-term prognosis of this disease”.* |
| **Responsiveness** | We need to continue to ensure that we are responsive to the needs of young people too, especially as new information emerges about the ways they are being harmed by COVID-19 that may be outside the ‘transmission’ concerns.  A formal mechanism for parents, caregivers, and young people to provide feedback must be developed in a way that is attentive to the capacities of an organisation or state. | Adjustments and revisions to policies based on new research findings on Long COVID in CYP were analysed. | Criticism on social media regarding delays in policy implementation or failure to adapt to the evolving pandemic was identified.    Example:  *“There's a UK political lobby group made up of all parliamentarians, that have heard moving evidence of the lived experience of adults and kids with #LongCovid”* |

# Appendix 2

## Table 3. Inclusion and Exclusion criteria for key Long COVID policies

|  | **Inclusion** | **Exclusion** |
| --- | --- | --- |
| **Participants** | 1. Healthcare Workers (HCWs)  2. AND employed  3. AND during the COVID-19 pandemic | 1. not HCWs  2. OR unemployed  3. OR before the COVID-19 pandemic |
| **Intervention** | Policies or guidelines specifically designed for:  1. HCWs management of patients suffering (or suspected to suffer) from post-acute coronavirus syndrome  2. OR to advise patients diagnosed with post-acute coronavirus syndrome | Policies or guidelines specifically designed to address:  1. Healthcare in response to the COVID-19 pandemic with no post-acute coronavirus syndrome (OR Long COVID) component |
| **Comparator** | N/A | |
| **Outcomes** | 1. Service delivery to respond to post- acute coronavirus syndrome  2. OR recommendations to respond to post-acute coronavirus syndrome | 1. No service delivery to respond to post- acute coronavirus syndrome  2. No recommendations to respond to post-acute coronavirus syndrome |
| **Study design** | N/A | |
| **Setting** | 1. United Kingdom (UK)  2. AND March 2020 - current | 1. Non-UK  2. OR pre-March 2020 |

# Appendix 3

## Boolean search terms used on Brandwatch™

(longcovid OR "long covid" OR "post-Covid syndrome" OR "post acute sequelae of SARS COV-2 infection" OR PASC OR "post-COVID-19 syndrome" OR "post-COVID conditions" OR "long COVID-19" OR "long-haul COVID-19" OR longcovidkids OR "long covid kid*" OR "long covid child*" OR "long covid teenag*") AND ((government OR gvnmt OR parliament OR parlement) OR (politic OR politics OR policy OR policies) OR (tory OR tories OR conservative OR conservatives OR lib OR liberal OR libs OR labour OR national OR democratic OR dems OR social OR socialist OR socialists OR "green party") OR (England OR UK OR Ireland OR Scotland OR Wales))

# Appendix 4

## Table 4. Policy timeline re. Long COVID (2020 - 2022)

| **Ref** | **Name/Title** | **Date of first**  **publication** | **Type** | **Source** | **Link** | **Target**  **Audience** |
| --- | --- | --- | --- | --- | --- | --- |
| 1 | COVID-19: paediatric surveillance | 27/04/2020 | Guidance | Public Health England (PHE) | <https://www.gov.uk/guidance/covid-19-paediatric-surveillance> | Healthcare providers |
| 2 | NHS to offer ‘Long  COVID’ sufferers help at specialist centres | 07/10/ 2020 | News | NHS | <https://www.england.nhs.uk/2020/10/nhs-to-offer-long-covid-help/> | Healthcare providers, patients |
| 3 | A dynamic review of the evidence around ongoing COVID19 symptoms  (often called Long COVID) | 15/10/ 2020 | Guidance | NIHR  (National Institute for  Health research) | <https://evidence.nihr.ac.uk/themedreview/living-with-covid19/#Resources> | Patients,  health care  providers, researchers |
| 4 | COVID-19 rapid guideline: managing the long-term effect of COVID-19 | 18/12/ 2020 | Guidance | NICE | <https://www.nice.org.uk/guidance/NG188> | Healthcare providers and  patients |
| 5 | NIHR announces 18.5 million GBP awarded to new research projects to understand and treat Long COVID | 18/02/ 2021 | News | NIHR | <https://www.nihr.ac.uk/news/ps185-million-awarded-new-research-projects-understand-and-treat-long-covid> | Healthcare providers, researchers |
| 6 | NIHR announces  second 20 million GBP long COVID funding call | 25/03/ 2021 | News | NIHR | <https://www.nihr.ac.uk/news/nihr-launches-second-ps20-million-long-covid-funding-call> | Healthcare providers, researchers |
| 7 | Long COVID call 2021: Guidance for  applicants | 25/03/ 2021 | News | NIHR | <https://www.nihr.ac.uk/documents/long-covid-call-2021-guidance-for-applicants/27341> | Healthcare providers, researchers |
| 8 | COVID-19 mental health and wellbeing recovery action plan | 27/03/ 2021 | Policy | Dept of Health &  Social  Care | <https://www.gov.uk/government/publications/covid-19-mental-health-and-wellbeing-recovery-action-plan/covid-19-mental-health-and-wellbeing-recovery-action-plan> | Health Care Providers |
| 9 | National  guidance for  post- COVID syndrome assessment clinics | 26/04/ 2021 | Guidance | NHS | <https://www.england.nhs.uk/coronavirus/wp-content/uploads/sites/52/2020/11/C1248-national-guidance-post-covid-syndrome-assessment-clinics-v2.pdf> | Healthcare providers |
| 10 | Coronavirus  (COVID-19): long term health effects | 30/04/ 2021 | Guidance | UK  Health  Security Agency | <https://www.gov.uk/government/publications/covid-19-long-term-health-effects/covid-19-long-term-health-effects> | Patients  and health care  providers |
| 11 | Long COVID Plan 21/22: 10 key next step to support those suffering from LC | 05/06/2021 | Policy | NHS | <https://www.england.nhs.uk/coronavirus/wp-content/uploads/sites/52/2021/06/C1312-long-covid-plan-june-2021.pdf> | Healthcare providers |
| 12 | Enhanced service specification  (Long COVID  2021/2022) | 21/06/2021 | Policy | NHS | <https://www.england.nhs.uk/wp-content/uploads/2021/06/C1313-ess-long-covid-21-22.pdf> | Healthcare providers |
| 13 | Up to one in  three people who have had COVID-19 report Long COVID  symptoms | 24/06/2021 | News | NIHR | <https://www.nihr.ac.uk/news/one-three-people-who-have-had-covid-19-report-long-covid-symptoms> | Healthcare providers |
| 14 | People with five or more symptoms more likely  to  develop Long COVID | 16/07/2021 | News | NIHR | <https://www.nihr.ac.uk/news/people-five-or-more-symptoms-more-likely-develop-long-covid> | Healthcare providers |
| 15 | First findings from world’s largest study on  Long COVID in children and young people (cites the: Non-hospitalised  Children & young people (CYP) with Long  COVID  (The CLoCk  Study)) | 02/09/2021 | News | NIHR | <https://www.nihr.ac.uk/news/first-findings-worlds-largest-study-long-covid-children-and-young-people> | Healthcare providers |
| 16 | Over a third of COVID-19 patients diagnosed with at least one Long COVID symptom | 30/09/2021 | News | NIHR | https://www.nihr.ac.uk/news/over-a-third-of-covid-19-patients-diagnosed-with-at-least-one-long-covid-symptom/28799 | Healthcare providers |
| 17 | Winter workforce preparedness | 01/12/2021 | Policy | NHS | <https://www.england.nhs.uk/winter/winter-workforce-preparedness/> | Healthcare providers |
| 18 | Find help and support if you have Long COVID | 24/12/2021 | Guidance | Department of Health &  Social Care | <https://www.gov.uk/guidance/find-help-and-support-if-you-have-long-covid> | Patients |
| 19 | Long-term effect  of  coronavirus (Long COVID) | 19/01/2022 | Guidance | NHS | <https://www.nhs.uk/conditions/covid-19/long-term-effects-of-covid-19-long-covid/> | Patients |
| 20 | Lung abnormalities found in Long COVID patients with  breathlessness | 29/01/2022 | News | NIHR | <https://www.nihr.ac.uk/news/lung-abnormalities-found-in-long-covid-patients-with-breathlessness/29798> | Healthcare providers |
| 21 | Guidelines for supporting our NHS people affected by LC | 01/02/2022 | Guidance | NHS | <https://www.england.nhs.uk/wp-content/uploads/2022/01/C1506-guidelines-for-supporting-our-nhs-people-affected-by-long-covid.pdf> | Healthcare providers |
| 22 | New definitions for Long COVID developed with patients and carers. (cites the: Non-hospitalised  Children & young people (CYP) with Long COVID (The  CLoCk Study)) | 08/02/2022 | Guidance | NIHR | <https://www.nihr.ac.uk/news/new-definitions-long-covid-developed-patients-and-carers> | Healthcare providers |
| 23 | CLoCK protocol | June 2020  to  Dec  2023 | Research | NIHR (funded) | <https://assets.publishing.service.gov.uk/government/uploads/system/uploads/attachment_data/file/977177/Children_and_young_people_with_Long_Covid__CLoCK_.pdf> | Healthcare providers |
| 24 | Prevalence of  ongoing  symptoms  following  coronavirus (COVID-19) infection in the UK | Mont-hly  from April 2021 to at least Feb  2022 | News | Office for  National Statistics | <https://www.ons.gov.uk/peoplepopulationandcommunity/healthandsocialcare/conditionsanddiseases/bulletins/prevalenceofongoingsymptomsfollowingcoronaviruscovid19infectionintheuk/1april2021> | Healthcare providers |
